# Results of the pToWin Study: Using the pCONUS Device for the Treatment of Wide-Neck Intracranial Aneurysms

**DOI:** 10.3390/jcm11030884

**Published:** 2022-02-08

**Authors:** Marta Aguilar Pérez, Hans Henkes, Wiebke Kurre, Carlos Bleise, Pedro Nicolás Lylyk, Javier Lundquist, Francis Turjman, Hanan Alhazmi, Christian Loehr, Stephan Felber, Hannes Deutschmann, Stephan Lowens, Luigi Delehaye, Markus Möhlenbruch, Jörg Hattingen, Pedro Lylyk

**Affiliations:** 1 Neuroradiological Clinic, Katharinenhospital Stuttgart, 70174 Stuttgart, Germany; hhhenkes@aol.com; 2 Medical Faculty, University of Duisburg-Essen, 45147 Essen, Germany; 3 Department of Radiology and Neuroradiology, Klinikum Passau, 94032 Passau, Germany; wiebke.kurre@gmx.de; 4 Department of Interventional Neuroradiology, Clínica La Sagrada Familia, Buenos Aires C1426EOB, Argentina; cbleise@lylyk.com.ar (C.B.); ivan@lylyk.com.ar (P.N.L.); jlundquist@lylyk.com.ar (J.L.); taras@lylyk.com.ar (P.L.); 5 Department of Interventional Neuroradiology, Hôpital Pierre Wertheimer, 69500 Lyon, France; francis.turjman@chu-lyon.fr (F.T.); h.alhazmi989@gmail.com (H.A.); 6 Department of Radiology and Neuroradiology, Klinikum Vest Knappschaftskrankenhaus Recklinghausen, 45657 Recklinghausen, Germany; christian.loehr@klinikum-vest.de; 7 Department of Diagnostic and Interventional Radiology and Neuroradiology, Gemeinschaftsklinikum Mittelrhein, 56068 Koblenz, Germany; stephan.felber@gk.de; 8 Department of Radiology, Clinical Division of Neuroradiology, Vascular and Interventional Radiology, Medical Universtity Graz, 8063 Graz, Austria; hannes.deutschmann@klinikum-graz.at; 9 Department of Radiology, Klinikum Osnabrück, 49076 Osnabruck, Germany; stephan.lowens@klinikum-os.de; 10 Unit of Interventional Neuroradiology, Ospedale San Giovanni Bosco, 80144 Napoli, Italy; gdelehaye@yahoo.it; 11 Department of Neuroradiology, Heidelberg University Hospital, 69120 Heidelberg, Germany; markus.moehlenbruch@med.uni-heidelberg.de; 12 Institute of Neuroradiology, KRH Klinikum Nordstadt, 30167 Hannover, Germany; joerg.hattingen@krh.eu

**Keywords:** coiling, endovascular treatment, intracranial aneurysms, pCONUS, stent-assisted coiling, wide-neck bifurcation aneurysms

## Abstract

Coil embolization has become a well-established option for the treatment of intracranial aneurysms. Yet, wide-neck bifurcation aneurysms (WNBAs) remain a challenge. The pCONUS is the first generation of a stent-like implant for the bridging of WNBAs to enable coiling. The pToWin study was a prospective, single-arm, multicenter study conducted to analyze the safety and efficacy of the pCONUS in the treatment of WNBAs. The primary effectiveness endpoint was the rate of adequate occlusion of the aneurysm at 3–6 and 7–12 months. The primary safety endpoint was the occurrence of major ipsilateral stroke or neurological death during the follow-up. A total of 115 patients were included. Aneurysm locations were the middle cerebral artery in 52 (45.2%), the anterior communicating artery in 35 (30.4%), the basilar artery in 23 (20%), the internal carotid artery terminus in three (2.6%), and the pericallosal artery in two (1.7%) patients. Treatment was successfully performed in all but one patient. The morbi-mortality rate was 1.9% and 2.3% at 3–6 and 7–12 months, respectively. Of the aneurysms, 75.0% and 65.6% showed adequate occlusion at 3–6 and 7–12 months, respectively. pCONUS offers a safe and reasonably effective treatment of WNBAs, demonstrated by acceptable adequate aneurysm occlusion and low rates of adverse neurologic events.

## 1. Introduction

The majority of intracranial aneurysms (IAs) are located at one of the bifurcation points of the Willis polygon, and are considered to be challenging for endovascular treatment since they usually have a wide neck [1]. A growing number of dedicated devices have been engineered to deal with the troublesome anatomy of wide-neck bifurcation aneurysms (WNBAs), including the pCONUS device (Phenox, Bochum, Germany). The first generation of this device consisted of a stent-like support for the proximal vessel (shaft), with four distal clover-shaped petals creating an effective scaffold for coiling, reinforced by a net of six nylon fibers in the center of the construct to ensure adequate neck support [2,3]. In the second generation, the nylon net is absent, and the distal crown (with two more petals) emanates from a central spur. This spur creates an articulation zone between the distal crown and the stent shaft, improving flexibility. Additionally, the shaft is shorter, reducing the amount of metal in the parent vessel [4].

Here, we present the results of the pCONUS treatment of wide-neck intracranial aneurysms (pToWin) study, a clinical trial of the first generation of the pCONUS device. The purpose of this study was to evaluate the safety and effectiveness of the pCONUS as a permanent implant to assist the coil occlusion of WNBAs. The primary effectiveness endpoint was an angiographic evaluation that demonstrated adequate occlusion (either complete occlusion or neck remnant) of the target aneurysm at 3–6 and 7–12 months, assessed by an independent Core Lab. The primary safety endpoint was the occurrence of major ipsilateral stroke or neurologic death during the follow-up period.

## 2. Materials and Methods

### 2.1. Industry Support

Our trial was supported by Phenox GmbH. The funding source provided financial support to the participating sites based on patient enrollment in the trial. Additional support from the funding source included frequent monitor visits to verify source data and ensure compliance with the protocol.

Except for one author (H.H.), a shareholder of the sponsor, the investigators had no financial conflict of interest during enrollment.

### 2.2. Study Design and Participants

The pToWin study was a prospective, multicenter, single-arm, non-interventional post-market clinical follow-up study focused on treating WNBAs with the pCONUS device. Between September 2015 and August 2018, a total of 115 patients from 10 neurovascular centers were prospectively enrolled. Each participating investigator was required to have performed at least five proctored pCONUS administrations prior to entry into the study, which was designed, conducted, recorded, and reported in compliance with ISO 14155:2012 and ICH GCP. The institutional review board (or ethics committee) of each institution approved the study protocol and informed consent form. Written informed consent was obtained prior to entry into the study from all patients.

Main patient inclusion criteria were the presence of a WNBA arising from the terminal internal carotid artery (ICA terminus), anterior communication artery (AcomA), middle cerebral artery (MCA), or basilar artery (BA), with a fundus offering enough space for the safe deployment of the device crown. Patients were excluded if they had any of the following: intracranial hemorrhage apart from the target aneurysm, ischemic stroke, myocardial infarction, major surgery within the last 30 days, a previously placed stent or an intra-aneurysmal implant beside coils, known coagulopathy or allergy to the components of the device, evidence of active infection, or significant stenosis within the vascular access. Patients with contraindications to the contrast media required for angiography were also excluded, as were patients with a progressive neurological disorder, an arteriovenous malformation, fistula, or another aneurysm requiring treatment within the following six months. Acutely ruptured aneurysms were not excluded, unless the patient was clinically severely affected (Hunt and Hess grade IV-V) and/or there was evidence of severe vasospasm, parenchymal hemorrhage, or subdural hematoma.

### 2.3. Endovascular Treatment

All procedures were performed according to the established clinical routine [5]. Angiographic runs and image documentation during the procedure were performed according to the operator’s usual practice. Final angiographic runs included magnified images of the aneurysm in working projections, clearly depicting the vessel branches arising from the aneurysm and the aneurysm neck itself, and an overview run allowing for the assessment of all peripheral branches of the dependent vessel territory.

Pre-, intra-, and post-operative antiplatelet therapy (APT) was managed separately in each center. Antiplatelet activity testing was not required in the study protocol. Patients were temporarily administered therapeutic heparinization, which was discontinued at the end of the procedure. Activated clotting times were checked prior to and during treatment.

Appropriate device sizing was determined based on 2D and 3D digital subtraction angiography (DSA). The following microcatheters were used during the procedures: Excelsior SL-10 or Trevo18 Pro (Stryker, Fremont, CA, USA), Prowler Select Plus or Rapid Transit (Codman Neurovascular, Raynham, MA, USA), Marksman or Rebar18 (Medtronic, Irvine, CA, USA), or Vasco (Balt, Irvine, CA, USA).

### 2.4. Data Collection and Follow-Up

Each center completed a patient file with the following data:

- Patient demographic data and relevant comorbidities.
- Aneurysm characteristics, such as type, status at presentation, size, and location.
- Procedural characteristics, such as number and size of pCONUS used, antiplatelet medication, device deployment success, and complications during or after the procedure.

Patients underwent a neurologic examination according to the National Institute of Health Stroke Scale Score (NIHSS) and the modified Rankin Score (mRS) before treatment, at hospital discharge, at 3–6 months, and at 7–12 months, according to site-specific standards. The Hunt and Hess Score (HH) was also recorded for patients with ruptured aneurysms. Vascular imaging at follow-up was performed according to the operator’s usual practice (digital subtraction angiography—DSA, magnetic resonance angiography—MRA, computed tomographic angiography—CTA). Data from retreatment procedures were also collected. Aneurysm occlusion was rated using the Raymond–Roy Scale [6]: class I, complete occlusion; class II, neck remnant; and class III, residual aneurysm.

Data reported by the study sites were source data verified by dedicated local monitors. All image material was reviewed by an independent Core Lab reviewer for the assessment of aneurysm occlusion status and complications visible on image material.

### 2.5. Study Endpoints

The primary endpoints of this study were as follows:

- Effectiveness: The rate of adequate occlusion (complete or neck remnant) of the target aneurysm at 3–6 and 7–12 months was met.
- Safety: The incidence of major ipsilateral stroke (an increase of four or more points according to the basal NIHSS) or neurological death within the follow-up phase.

Several secondary endpoints were also defined by the evaluation of treatment feasibility and technical success, the description of intra- and post-procedural complications, as well as clinical and anatomical outcomes at follow-up.

### 2.6. Statistical Analysis

The primary analysis set of all primary and secondary endpoint study outcomes was the per-protocol population (PP), under which data from all enrolled patients were analyzed, including those who met all eligibility criteria and underwent embolization with the pCONUS device with at least one pCONUS implanted. A secondary analysis set of all study outcomes was intent-to-treat (ITT), under which data from all enrolled patients were analyzed irrespective of the treatment actually delivered and of each patient’s eligibility.

Discrete variables were summarized using frequency and percentage. Continuous variables were summarized using the number of observations (N), mean, standard deviation (SD), median, and minimum and maximum values. Statistical analyses were performed using SPSS Statistics version 27 (IBM, Armonk, NY, USA).

## 3. Results

### 3.1. Patient and Aneurysm Population

Between September 2015 and August 2018, 115 patients (77 (66.9%) females) were enrolled in ten centers across five countries (ITT population). In one patient, a pCONUS embolization was attempted but not implanted. A total of six patients were considered to be non-eligible after signing informed consent and after the pCONUS implant attempt had begun. In total, 108 patients met all eligibility criteria and underwent pCONUS treatment (PP population).

Baseline patient characteristics (ITT population), including demographics and medical history, are shown in Table 1. Age ranged from 30 to 80 years (mean: 60.0±10.0 years). Notably, 48 (41.7%) patients were hypertensive, and 40 (34.8%) patients were current or former smokers; previous stroke (including subarachnoid hemorrhage and intracerebral hematoma) was reported in 25 (21.7%) patients.

All aneurysms but one were unruptured (99.1%), and 14 (12.2%) had already been previously treated. Among the recanalized aneurysms, initial treatment was coiling in 11 (9.6%) and clipping, wrapping, or stent-assisted coiling in one patient each (0.9%). The majority of the aneurysms were located at the MCA bifurcation (*n* = 52), followed by AcomA (*n* = 35) and basilar tip (*n* = 23). Aneurysm dimensions were based on the evaluation of the treating investigator and classified as small (<7mm, 68.7%), medium (7–12mm, 27.8%), large (13–24mm, 3.5%), and giant (≥25mm, 0.0%) [7]. The mean dome width was 6.8mm, the mean dome height was 6.2mm, and the neck width was 4.9mm. A mean dome-to-neck ratio of 1.4 was calculated from these results. In 71.1% of aneurysms, a dome-to-neck ratio <1.5 was observed, and could thus be classified as wide-neck aneurysms. The baseline characteristics of the target aneurysms are presented in Table 2.

Antiplatelet activity testing was performed in 86 (74.8%) patients, and was not analyzed. Almost all ITT patients (94.8%) received dual APT as recommended. Three (2.6%) patients received a single APT, one (0.2%) patient received triple antiplatelet medication (ASA, clopidogrel, and ticagrelor), while for two (1.7%) patients, no medication was documented. The duration of APT varied among centers. The most common regimen was dual APT for up to three months, continuing with single APT beyond twelve months (31.3%).

### 3.2. Procedural Characteristics and Treatment Feasibility

Treatment with the pCONUS device was successfully performed in 114 out of 115 (99.1%) patients (ITT population). In one (0.9%) patient, the device was implanted and navigated with a second microcatheter to coil the aneurysm sac. During coiling, the device migrated. Nevertheless, the deployed coils appeared to remain correctly positioned in the aneurysm. Thus, the treating physician decided to remove the implanted pCONUS without replacing it. The aneurysm sac was further packed with coils until adequate occlusion was achieved. Post-procedurally, the Core Lab confirmed that the dome height/fundus of the aneurysm did not offer enough space for the pCONUS crown to deploy.

In four (3.5%) patients, a second pCONUS device was used to treat the aneurysm. The reasons for requiring a second pCONUS were either an initial wrong sizing of the device (*n* = 3) or a wrong device variation was initially chosen. In addition, there was one case in which two different pCONUS devices were implanted to treat the aneurysm successfully. The coverage of the aneurysm neck with a single pCONUS seemed to be insufficient. Thus, following the implantation of a first pCONUS without nylon cross, a second pCONUS with a nylon cross was released coaxially. Finally, the aneurysm sac was catheterized through both pCONUS devices and successfully coiled.

At the end of the procedure, complete occlusion was achieved in 57/115 (49.6%) aneurysms, whereas 23/115 (20.0%) aneurysms showed a neck remnant, and 35/115 (30.4%) aneurysms showed residual perfusion at the end of the procedure.

### 3.3. Patient Disposition at Follow-Up

The 3–6-month visits were scheduled at a mean of 140 days (median: 153 days, range: 22–215 days). The 7–12-month visits were scheduled at a mean of 419 days (median: 385 days, range: 217–1087 days) post-intervention.

**Figure 1:**
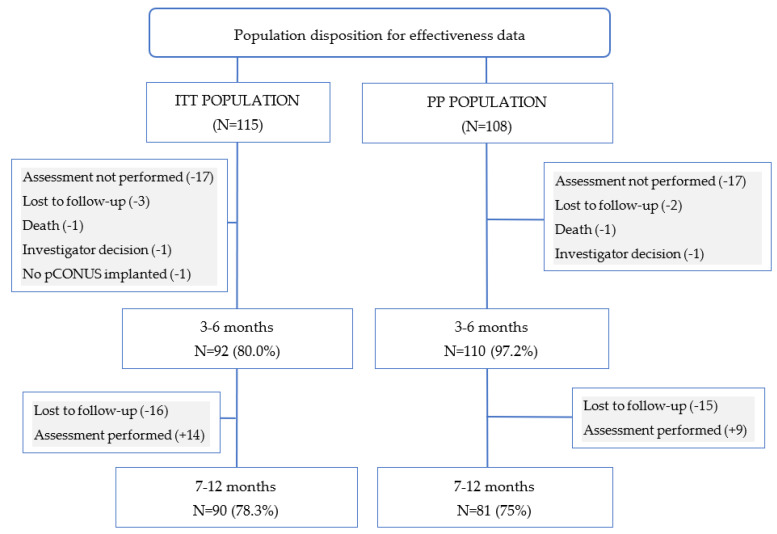
Population disposition for effectiveness at 3–6- and 7–12-month follow-ups. “Lost to follow-up” means the centers could not contact the patients despite using different techniques (e.g., letter, phone call, email), so the patients exited the study. “Exam not available” means the patient did not attend this follow-up, but may have attended a later one. PP: per-protocol population; ITT: intent-to-treat population.

Figure 1 shows the disposition of clinical data collected from ITT and PP patients. Of the ITT patients, 95.7% (110/115) had the 3–6-month safety data available, and 81.7% (94/115) had the 7–12-month safety data available. Of the PP patients, 97.2% (105/108) had the 3–6-month safety data available, and 81.5% (88/108) had the 7–12-month safety data available.

**Figure 2:**
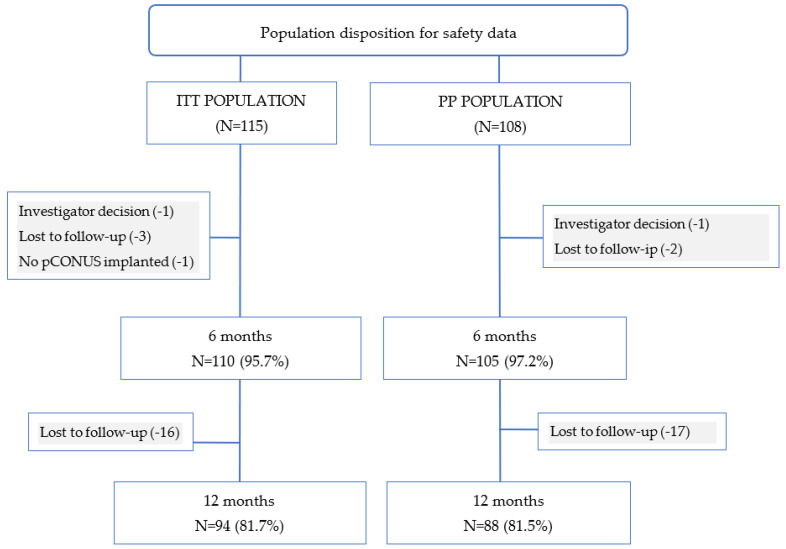
Population disposition for safety at 3–6- and 7–12-month follow-ups.

Figure 2 shows the disposition of the angiographic data collected from ITT and PP patients. Angiographic assessments were performed for 80.0% (92/115) of ITT patients at 3–6 months post-procedure and 78.3% (90/115) of ITT patients at 7–12 months. The results for angiographic assessments in the PP population are very similar to the ITT population, with 80.6% (87/108) of 3–6-month visits and 72.2% (78/108) of 7–12-month visits performed due to the PP selection criteria.

The primary effectiveness endpoint was the rate of adequate aneurysm occlusion at the 3–6- and 7–12-month angiographic follow-up visits. Of the aneurysms, 75.0% (69/92) and 65.6% (59/90) met the primary effectiveness endpoint of adequate occlusion at the 3–6- and 7–12-month follow-ups, respectively. Residual aneurysm was observed in 23/92 (25%) at 3–6 months and 31/90 (34.4%) at 7–12 months.

An analysis of the evolution of the occlusion status over the follow-up period of the study is shown in Table 3. While for 26.7% of ITT patients (N = 90 at second follow-up), the aneurysm remained completely occluded (class I) throughout the study, in 8.9% the occlusion status changed from either neck remnant (class II) or residual aneurysm (class III) to complete occlusion, and in 14.4% the status changed from complete occlusion/neck remnant to residual aneurysm. In four patients (4.3%), a total of seven retreatments were performed. Two patients underwent multiple retreatments in different follow-up periods of the study.

The secondary effectiveness endpoint was the rate of intra-procedural technical complications, such as the placement of the pCONUS in the desired position and its correct opening and detachment. Based on Core Lab data, 98.3% (113/115) of the implanted devices were deployed at the desired location. In one case, the pCONUS was placed too low in the aneurysm neck and, in another one, the crown of the pCONUS was partially placed in the parent vessel. However, in all cases, the coiling of the aneurysm sac was possible without obliterating the side branches.

### 3.4. Safety Endpoints

The primary safety endpoint was the rate of major stroke (ischemic or hemorrhagic) or neurological death during the follow-up period. Two patients experienced a major stroke during the first 6 months post-intervention, one of which resulted in death. There were no additional events observed in the second 6 months post-intervention. Thus, at the 3–6- and 7–12-month follow-ups, respectively, 1.9% (2/105) and 2.3% (2/88) of the patients met the primary safety endpoint. One patient experienced a traumatic SAH 152 days after treatment, followed by intracerebral hematoma four days later. This event was classified as not procedure-related, and was unlikely to be related to the pCONUS. The second patient experienced a massive SAH caused by an aneurysm rupturing 30 min after an uneventful treatment of an AcomA aneurysm with the pCONUS device. External ventricular drainage was inserted, and a decompressive craniectomy was performed. However, the patient developed refractory intracranial hypertension and died 3 days after the intervention. This event was classified as definitively related to the intervention but unrelated to the pCONUS, as no contrast extravasation was observed in the DSA.

Secondary safety endpoints were assessed based on the angiographic results, and these are summarized in Table 4. Six events were observed peri-procedurally. In two (1.9%) patients, the aneurysm was perforated with the pCONUS device during the intervention, and thromboembolic events were reported in four (3.7%) patients. During the follow-up period, a small subarachnoid hemorrhage was found in one (1.0%) patient of the PP population on day 3 post-intervention, which was classified as procedure-related. Additionally, one (1.0%) rupture of the treated aneurysm was observed within the 3–6-month follow-up period. On day 203, this patient experienced a subarachnoid hemorrhage the day before a planned retreatment for the aneurysm while being already hospitalized. The aneurysm was retreated as planned the next day. No additional ruptures of the treated aneurysms were observed during the 7–12-month period. Within 3–6 months after the intervention, one patient suffered an ischemic stroke, while within the 7–12-month period, a second ischemic stroke occurred (cumulative incidence 2.3%). Both ischemic strokes were considered to be minor.

A total of 60 adverse events were reported during the study in 42 (36.5%) of the 115 patients. Of these 60 events, 33.3% (20/60) were classified as serious (serious adverse events—SAE) and occurred in 18 patients (18/115, 15.7%). The classification of adverse events as serious was based on ISO 14155:2012. One (0.9%) patient died. Twelve (10.4%) patients were hospitalized, or their hospitalization was prolonged. In three (2.6%) patients, a medical or surgical intervention was performed to prevent life-threatening illness or injury/permanent impairment to a body structure or function, and four (3.5%) patients showed permanent impairment of a body structure or function.

Pre-procedural mRS scores were documented for 103 patients and for 90 patients at the 7–12-month post-procedure follow-up. Before the intervention, most patients (95.1%) had a 0-2 mRS. This percentage decreased slightly to 94.4% at the end of the study. Table 5 shows the distribution of mRS scores during the study.

## 4. Discussion

WNBAs have typically been considered to be challenging for endovascular treatment. Classically, various stent configurations have been described to protect the efferent branches of the bifurcation and allow the coiling of the aneurysm [8]. However, stent placement requires crossing the aneurysm neck and the selective catheterization of at least one of the efferent branches, which can be technically demanding because of the angle of disposition related to the parent vessel. The “waffle cone technique” was described as an alternative to the different stent configurations [9], but the stents used were not optimized for this purpose, and were far from ideal. In recent years, numerous stent-like devices have specifically been designed to deal with the troublesome anatomy of WNBAs, including pCONUS and pCANVAS (Phenox, Bochum, Germany), PulseRider (Pulsar Vascular, CA, USA), eCLIPS (Evasc Medical Systems, Vancouver, Canada), and Barrel (Medtronic/Covidien, CA, USA). At the time of this writing, only pCONUS, PulseRider, and eCLIPS are still available on the market for the endovascular treatment of intracranial aneurysms. Apart from these stent-like devices, extrasaccular flow diversion and intrasaccular flow disruptors, such as Woven EndoBridge (WEB; MicroVention, Aliso Viejo, CA, USA) or, more recently, Contour (Cerus Endovascular, Fremont, CA, USA), have emerged as an alternative treatment of WNBAs.

At the time of study start, the pCONUS was a novel device. The first case series included 28 consecutive patients in a single-center study and showed excellent feasibility and safety of the device [9]. Meanwhile, several retrospective studies on pCONUS became available, but these studies were limited to small cohorts [10,11,12,13,14,15,16]. Recently, Krupa et al. [17] summarized the pCONUS literature in a systematic review and meta-analysis, with a total of eight studies (198 patients with 200 aneurysms) included. Immediately after the procedure, adequate occlusion was observed in 79.7% of patients, increasing further to 84.0% at the six-month mark. Intraprocedural complications were observed in 17.3% of patients, with the most frequent event being thromboembolic (12.1% of all procedures).

The pToWin study represents the only prospective study to date to evaluate the safety and efficacy of the first generation of the pCONUS device in treating WNBAs. Ten international neurovascular centers contributed 115 patients with 115 aneurysms between September 2015 and August 2018. This study collected data from one-year clinical follow-ups (safety) in 81.7% of patients and one-year anatomical follow-ups (efficacy) in 78.3% of patients.

Data from the pToWin study demonstrate that the pCONUS device can be implanted with a high technical success rate. All devices opened correctly, detached properly, and occluded the aneurysm without side branch obliteration. The rate of intraprocedural complications was 5.6%, including two (1.9%) experiencing aneurysm perforation and four (3.7%) thromboembolic events, which is a significant improvement over Krupa and colleagues’ meta-analysis (17.3%), and is comparable with the rate of intraoperative complication reported during PulseRider implantation [18], where a total of five out of sixty-three (7.9%) complications were reported, including two (3.1%) aneurysm ruptures, two (3.1%) thromboembolic events, and one (1.6%) vessel dissection. However, adequate occlusion could not be achieved in 30.4% of the aneurysms after treatment. A possible explanation may be the inclusion of aneurysms with steep angles between the parent vessel and the aneurysm. In these angulated aneurysms, one petal of the pCONUS connects with the aneurysm wall near the base, while a gap at the inside of the angle remains, since the petals deploy nearly orthogonally from the device stem. This anatomical situation may prevent complete occlusion or lead to coil protrusion, and was already described by Ulfert et al. in their series of 22 aneurysms treated with the pCONUS [15]. The use of the second generation of the device, which allows the crown to articulate and accommodate angles between the parent vessel and the aneurysm, may improve initial angiographic results.

In the pToWin study, 75.0% (69/92) and 65.6% (59/90) of the aneurysms met the primary effectiveness endpoint of adequate occlusion at the 3–6- and 7–12-month follow-ups, respectively. This occlusion rate is lower than that achieved in the meta-analysis of Krupa et al., and even lower compared with the occlusion rate reported in the one-year follow-up of the ANSWER trial using the PulseRider device [19], where the rate of adequate occlusion improved from 88.0% at six months to 90.0% at twelve months, and no recanalization was observed. A possible explanation of our low occlusion rate at first and second follow-up may be, as mentioned above, that already 30.04% of the aneurysms were initially not adequately occluded. This fact, added to the need for dual antiplatelet therapy after treatment, may avoid further thrombosis of the aneurysms and propitiate recanalization. The use of more recently developed low-thrombogenic implants (pCONUS-HPC) under single antiplatelet therapy (SAPT) may help the aneurysm to thrombose. In contrast with pCONUS or PulseRider, the eCLIPs bridges the aneurysm neck, allowing coiling, but also combining flow diversion properties that contribute to aneurysm closure. Recently, De Vries et al. [20] showed comparable efficacy and safety, despite the high incidence of device deployment failure (24.0% of procedures) by Chiu et al. in their initial experience [21].

Since their introduction, endoluminal flow diverters have been widely accepted as a treatment option for intracranial aneurysms. However, the concept of flow diversion in aneurysms located at the bifurcation is difficult, as at least one efferent branch must be covered, which may lead to increased complications and lower occlusion rates. Recently, one systematic review and meta-analysis regarding the use of endoluminal flow diverters in treating both sidewall and bifurcation aneurysms showed no significant differences in complication or occlusion rates between both groups [22]. This review included 35 studies with a total of 1,084 patients with 1,208 aneurysms (654 sidewall and 554 bifurcation aneurysms). The angiographic follow-up showed complete occlusion in 74.0% of the bifurcation aneurysms, with a complication rate of 20.4%.

Advances in the design of endoluminal devices have led to a greater interest in the development of endosaccular flow disruptors, which have the advantage that APT would not be required, nor would coverage of the normal side branches since, no parent vessel component is present. WEB is the most well known and most studied flow disruptor device. In a recently published systematic review and meta-analysis that included data from 963 aneurysms [23], the adequate occlusion rate at last follow-up was 83.3%, with a cumulative morbidity and mortality of 2.9% and 0.9%, respectively. The Contour device is the latest of this new generation of intrasaccular technologies. Although the experience with this device is still limited, the first human experiences have shown promising results. The longest series till now included eleven patients with eleven aneurysms [24]. After one year, they reported adequate occlusion in all aneurysms available for follow-up, with 55.6% having complete occlusion and 44.4% with small neck remnants. The compression of the WEB at follow-up can occur in up to 31.6% of cases [25]. Another series on the use of WEB reported a 46.7% retreatment rate for increasing the shortening of the device and distal location [26]. Bhogal et al. [27] reported the displacement of the Contour in one single case of their series of three patients, which was thought to be related to an inappropriate sizing and position of the device.

## 5. Limitations

This study has some limitations. First, pToWin is not a randomized trial, and safety and effectiveness cannot be directly compared with a control group. A second limitation is selection bias across the neurovascular centers and individual neurovascular practitioners. Similarly, periprocedural and follow-up management was at the institution’s discretion and was reflected in variations in platelet-function testing, antiplatelet therapy, and the scheduling of follow-up imaging. Finally, several patients were lost during the follow-up period, which might affect the overall occlusion rate.

## 6. Conclusions

In conclusion, the pToWin study demonstrates that the pCONUS device can be successfully and safely implanted, with moderate effectiveness and durability of the occlusion over time. Given that this type of neck-bridging device only allows for the coiling of a bifurcation or terminus aneurysm, and does not possess flow-diverter properties, the water hammer effect of blood entering the aneurysm neck and subsequent coil compaction are still a challenge. Therefore, it remains unclear if the pCONUS device offers a significant benefit over other treatment modalities.

## Figures and Tables

**Table 1:** Baseline demographics and medical history (ITT patients).

Variable	Results
Age (years)	60.0 ± 10.0 (30, 80–115)
Female	66.9% (77/115)
BMI	26.8 ± 5.6 (16.2, 52.4–115)
Medical history	
Previous stroke	21.7% (25/115)
Familial IAs	7.0% (8/115)
Atrial fibrillation	3.5% (4/115)
Myocardial infarction	5.2% (6/115)
Systemic hypertension	41.7% (48/115)
Coronary artery disease	3.5% (4/115)
Smoker	34.8% (40/115)
Alcohol abuser	7.0% (8/115)
Hyperthyroidism	1.7% (2/115)
Diabetes mellitus	7.8% (9/115)
Renal disease	4.3% (5/115)
Hyperlipidemia	13.0% (15/115)

BMI (body mass index). Summary statistics: continuous variables, mean ± SD (min, max-N); categorical, % (*n*/N).

**Table 2:** Aneurysm characteristics (ITT patients).

Characteristic	Results
Location	
MCA bifurcation	45.2% (52/115)
AcomA	30.4% (35/115)
BA tip	20.0% (23/115)
ICA terminus	2.6% (3/115)
Pericallosal artery	1.7% (2/115)
Ruptured status	
Ruptured (Core Lab)	0.9% (1/115)
Symptomatic	20.9% (24/115)
Previous treatment	
Coiling	9.6% (11/115)
Stent-assisted coiling	0.9% (1/115)
Clipping	0.9% (1/115)
Wrapping	0.9% (1/115)
Dimensions	
Dome width (mm)	6.8 ± 3.0 [6.0] (2.5, 18.0–114)
Dome height (mm)	6.2 ± 2.9 [6.0] (2.0, 22.6–115)
Neck width (mm)	4.9 ± 1.5 [4.7] (2.2, 9.7–115)
Dome-to-neck ratio	1.4 ± 0.6 [1.3] (0.4, 4.5–114)
<1.5	71.1% (81/114)
≥1.5	28.9% (33/114)
Size	
Small (<7 mm)	68.7% (79/115)
Medium (7–13 mm)	27.8% (32/115)
Large (13–25 mm)	3.5% (4/115)
Giant (≥25 mm)	0.0% (0/115)

Summary statistics: continuous variables, mean ± SD [median] (min, max–N); categorical, % (*n*/N).

**Table 3:** Transition in the effectiveness endpoint for ITT patients during the study based on the Raymond–Roy Scale.

	%	*n*/N
Stable class I	26.7%	24/90
Transitions to class I	8.9%	8/90
Stable class II	8.9%	8/90
Class I transitions to class II	13.3%	12/90
Class III transitions to class II	7.8%	7/90
Stable class III	20%	18/90
Class I transitions to class III	10%	9/90
Class II transitions to class III	4.4%	4/90

Class I, complete occlusion; class II, neck remnant; and class III, residual aneurysm.

**Table 4:** Secondary safety endpoint analysis (PP population).

Periprocedural	% (*n*/N)	
Thromboembolism	3.7% (4/108)	
Target aneurysm perforation	1.9% (2/108)	
Vessel perforation	0.0% (0/108)	
Dissection of access vessel	0.0% (0/108)	
**Follow-up**		
(Cumulative incidence)	**3–6 months**	**7–12 months**
Subarachnoid hemorrhage	1.0% (1/105)	1.1% (1/88)
Ruptured of the aneurysm	1.0% (1/105)	1.1% (1/88)
Ischemic stroke	1.0% (1/105)	2.3% (2/88)

**Table 5:** Modified ranking score (mRS) at pre-procedure, post-procedure, and at 3–6- and 7–12-month follow-ups (ITT population).

mRS	Pre-Procedure (N = 103)	Post-Procedure (N = 71)	3–6 Months (N = 95)	7–12 Months (N = 90)
0	81 (78.6%)	51 (71.8%)	68 (71.6%)	67 (74.4%)
1	10 (9.7%)	13 (18.3%)	17 (17.9%)	12 (13.3%)
2	7 (6.8%)	4 (5.6%)	5 (5.3%)	6 (6.7%)
3	4 (3.9%)	2 (2.8%)	2 (2.1%)	2 (2.2%)
4	1 (1.0%)	0 (0.0%)	2 (2.1%)	2 (2.2%)
5	0 (0.0%)	0 (0.0%)	0 (0.0%)	0 (0.0%)
6	0 (0.0%)	1 (1.4%)	1 (1.1%)	1 (1.1%)
Not assessed	12	44	15	4
mRS ≤ 2	98 (95.1%)	68 (95.8%)	90 (94.7%)	85 (94.4%)
mRS > 2	5 (4.9%)	3 (4.2%)	5 (5.3%)	5 (5.6%)

## Data Availability

The primary data presented in this study are available on request from the first author. The data are not publicly available due to patient privacy protection.
